# Retraction: Genome-wide association mapping of bread wheat genotypes for sustainable food security and yield potential under limited water conditions

**DOI:** 10.1371/journal.pone.0274147

**Published:** 2022-09-14

**Authors:** The PLOS ONE Editors

This article [1] was identified as one of a series of submissions for which we have concerns about authorship, competing interests, and peer review.

In addition, it came to light after publication that there are substantial text overlap and partial redundancy between this article [1] and a prior publication [2]. The two articles were under consideration during overlapping time periods, but the related work was not declared to *PLOS ONE* as is required by our policy on Submission and Publication of Related Studies. In response to this issue, the authors stated that the theme, aim, objectives, data analysis, parameters, and results were different between the two articles. The authors further noted that the two articles report analyses of the same genotypes, and the Population Structure sections of the article were similar because they are based on geographical distribution of the genotypes. The authors’ comments did not resolve the concerns about policy adherence and the degree of overlap between the two articles.

In light of these concerns, the *PLOS ONE* Editors retract this article. We regret that the issues were not addressed prior to the article’s publication.

All authors did not agree with the retraction.
